# A Normative Model of Serum Inhibin B in Young Males

**DOI:** 10.1371/journal.pone.0153843

**Published:** 2016-04-14

**Authors:** Thomas W. Kelsey, Amy Miles, Rod T. Mitchell, Richard A. Anderson, W. Hamish B. Wallace

**Affiliations:** 1 School of Computer Science, University of St Andrews, St Andrews KY16 9SX, United Kingdom; 2 School of Medicine, University of Edinburgh, Edinburgh EH16 4TJ, United Kingdom; 3 MRC Centre for Reproductive Health, University of Edinburgh, Edinburgh EH16 4TJ, United Kingdom; 4 Department of Haematology/Oncology, Royal Hospital for Sick Children, Edinburgh EH9 1LF, United Kingdom; University of Sydney, AUSTRALIA

## Abstract

Inhibin B has been identified as a potential marker of Sertoli cell function in males. The aim of this study is to produce a normative model of serum inhibin B in males from birth to seventeen years. We used a well-defined search strategy to identify studies containing data that can contribute to a larger approximation of the healthy population. We combined data from four published studies (n = 709) and derived an internally validated model with high goodness-of-fit and normally distributed residuals. Our results show that inhibin B increases following birth to a post-natal peak of 270 pg/mL (IQR 210–335 pg/mL) and then decreases during childhood followed by a rise at around 8 years, peaking at a mean 305 pg/mL (IQR 240–445 pg/mL) at around age 17. Following this peak there is a slow decline to the standard mature adult normal range of 170 pg/mL (IQR 125–215 pg/mL). This normative model suggests that 35% of the variation in Inhibin B levels in young males is due to age alone, provides an age-specific reference range for inhibin B in the young healthy male population, and will be a powerful tool in evaluating the potential of inhibin B as a marker of Sertoli cell function in pre-pubertal boys.

## Introduction

Inhibin B is a member of the transforming growth factor beta family of growth factors, and in males is secreted by the Sertoli cells of the testis that are located on the basement membrane of the seminiferous tubules, and nurture and support spermatogenesis. There is a well described negative feedback relationship, which becomes established around puberty [1, 2], with Follicle Stimulating Hormone (FSH) released from the anterior pituitary [3] under the control of gonadotrophin releasing hormone from the hypothalamus. Serum levels of inhibin B in adults have been shown to correlate positively with spermatogenesis [4–6]. Little is known about the significance of inhibin B levels in children [4, 7–10].

In a retrospective longitudinal study we have previously shown a potential role for inhibin B to predict fertility outcome in young males [11]. In adults the first study to report serum inhibin B as a marker of Sertoli cell function after chemotherapy was Wallace EM et al. (1997) [12], and a study by van Beek et al (2007) reported inhibin B as a marker of Sertoli cell damage in children treated for Hodgkin lymphoma [13]. However the interpretation of a marker of Sertoli cell function in young males requires an age-related normative model that quantifies the qualitative assessments of high neonatal inhibin B [14] followed by lower levels during childhood (although higher than that of females) [2, 15, 16]. Therefore the aim of this study was to identify and corroborate all studies containing data on the level of inhibin B and produce, and derive a normative model of inhibin B from birth to the age of seventeen years.

## Methods

**Fig 1:**
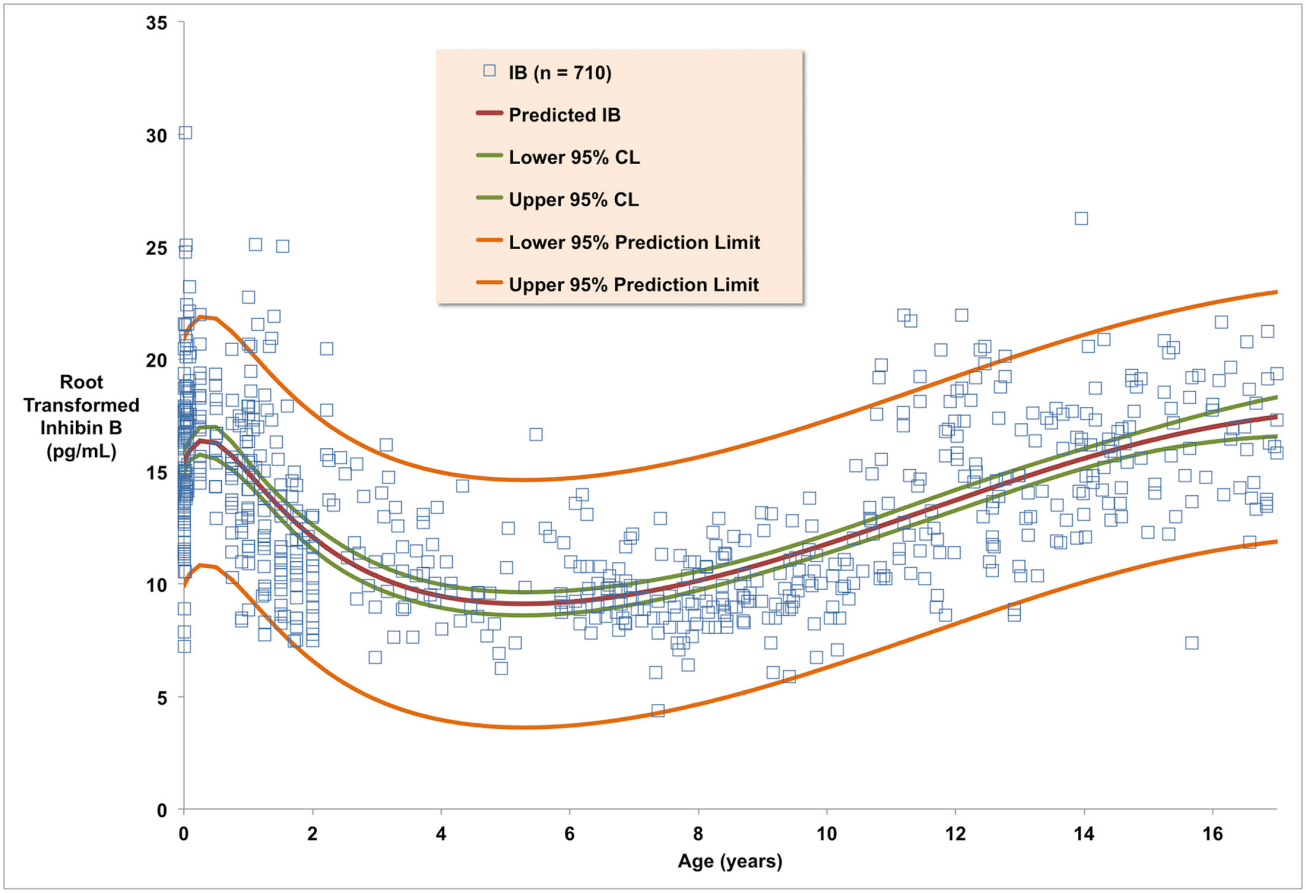
Root transformed Inhibin B levels. The red line is the model that best fits the 709 data points, which are indicated by blue squares. The model has r^2^ = 0.35, indicating that 35% of the variation in inhibin B levels in young males is due to age alone. The green lines represent the upper and lower 95% confidence limits for the predicted mean and the orange lines the upper and lower 95% prediction limits.

We searched two databases, Pubmed and Medline, for the search term inhibin B. The last search was performed in August 2015. Abstracts were studied, duplicates removed, and initial exclusion criteria applied. The exclusion criteria included, papers that were not written in English, those not containing humans and those not containing serum inhibin B levels. Full-text papers were then studied and further exclusion criteria applied to rule out studies not involving healthy male subjects, containing data that was not in an extractable form, not containing the correct age range, and using pubertal (i.e. Tanner) stage rather than age. The reference lists of selected studies were checked to identify further relevant studies. Data was mined from charts in the 4 identified studies (Table 1) using Web Plot Digitizer v3.9 [17] to convert datapoints into paired numerical values signifying age and inhibin B level (S1 Dataset). Each of the included studies used the same two-site ELISA assays with antibodies for Inhibin alpha and beta subunits developed by Groome et al [18]. The longitudinal data in each were treated as cross-sectional for model derivation. The resulting combined dataset (n = 709, median age 4.9 years, range 0–17 years) provides a sample that approximates the distribution of inhibin B levels in healthy human males for these ages (Fig 1).

**Table 1:** Summary of Inhibin B data. Year of publication first author and Pubmed ID for the papers used for data extraction, together with individual and combined study sizes and age statistics.

Year	1st Author	PMID	n	Median age	Age range
**1998**	**Andersson**	**9467591**	**133**	**1 year**	**0–2 years**
**2005**	**Radicioni**	**15757857**	**96**	**10.5 years**	**1.5–16.9 years**
**2002**	**Crofton**	**11874413**	**366**	**8.6 years**	**0–17 years**
**2006**	**Bergada**	**16849404**	**114**	**2.5 days**	**1–36 days**
**Total**			**709**	**4.9 years**	**0–17 years**

Approval was not required from an ethics committee or institutional review board since our research was limited to use of previously collected, non-identifiable data that has been published in peer reviewed journals which is specifically excluded from Research Ethics Committee review by the National Research Ethics Service guidelines of the UK Health Research Agency [19]. Patient data were anonymized and de-identified by the researchers involved in the published studies, prior to our analysis. Written informed consent was obtained from participants (or next of kin/caregiver in the case of children) for their clinical data to be published in the studies that provided the data that we extracted to derive our normative model. No patient identifiable information was available to us at any stage of our investigation.

We used an established methodology [20–23] to construct a normative model for Inhibin B. Inhibin B level at conception is known to be zero, so datapoints were added that forced models to go through this point (these additions were later deleted when assessing models by coefficient of determination and residual normality). Reference values for adults [24] were added to ensure correctness at ages above 17 years. To guard against sensitivity of the model at younger ages to the values imputed, model fitting following the random generation of adult values was repeated a total of ten times, with the imputed values included when assessing coefficient of determination and residual normality. Most studies of inhibin B report square-root adjusted values as levels are skew-normally distributed at given ages. Box-Cox analysis of our combined data showed an expected level of skewness that can be resolved by square-root adjustment—optimal lambda = 0.58 (95% CI 0.54–0.60)—which was performed before the model evaluation stage. TableCurve-2D (Systat Software Inc., San Jose, California, USA) was used to generate 255 mathematical models, which demonstrated predicted inhibin B level, 95% confidence limits for the model, and 95% prediction limits. The models were then evaluated for goodness of fit (using the r^2^ coefficient of determination) and normality of residuals (by comparing residual histograms to an idealised Gaussian distribution). The Akaike information criterion (the other common measure of goodness of fit for predictive models) was not calculated, since is equivalent to the r^2^ coefficient of determination for the linear least square regression models under consideration, with both measures being directly related to the residual sum of squares for a candidate model. We assessed candidate models for underfit (i.e low r^2^ due to insufficient complexity of the model in terms of number of model parameters) and overfit (i.e. models that are complex enough to obtain high r^2^ for the supplied data but which are also likely to have high error when generalised new data). To guard against models being selected by chance configuration of the data two forms of internal validation were implemented. 5-fold cross-validation was performed with residual mean training error for five randomly-chosen 80% subsets of the data compared to residual mean test error for the 20% subsets not used to fit the model. Bootstrap validation was performed on each candidate model by 2000 iterations of selecting with replacement ages and root-transformed pairs at random from the data, calculating the mean square error for each sample, and estimating the extra-sample prediction error by the mean of the bootstrap mean square errors.

## Results

The search for the term Inhibin B in Pubmed and Medline generated 1568 results, yielding 871 unique sources after removal of duplicates. The initial exclusion criteria were applied to the abstracts, resulting in 51 full-text studies being analysed and further exclusion criteria applied. Four papers were selected for quantitative synthesis in this study (Table 1).

**Fig 2:**
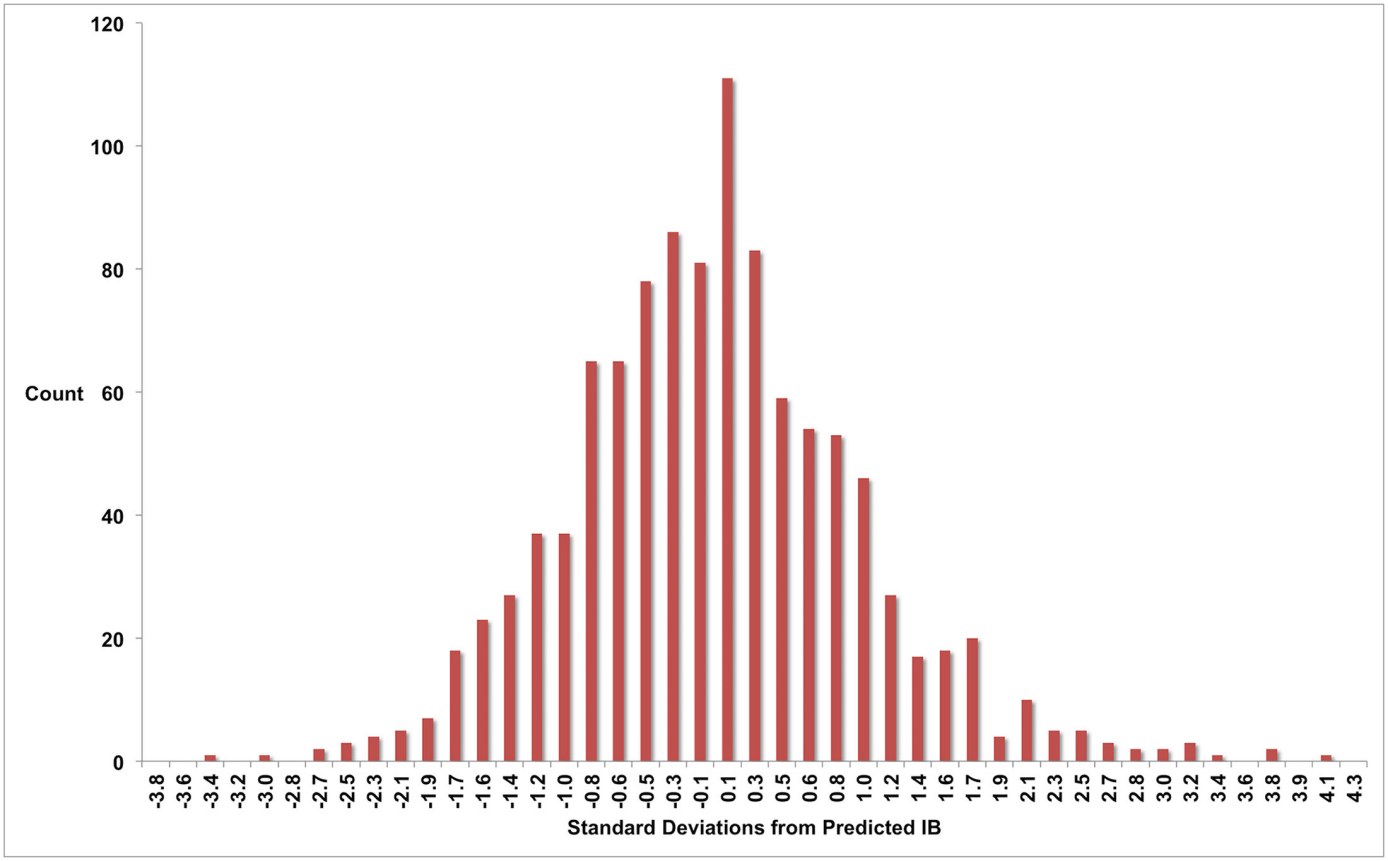
Model residuals. The residuals are the variations in root-adjusted observed values from the root-adjusted age-related mean value predicted by the model. The residuals have 97% goodness of fit to an ideal Gaussian curve.

The best performing class of models in terms of r^2^ and residual normality were rational polynomials. Models having fewer than 8 parameters displayed considerable underfit to the data, with r^2^ values over 10% lower than for the 8-parameter model. An 11-parameter model had higher r^2^ and lower residual mean error than the 8-parameter model, but this increase in model performance involved negative inhibin B values between conception and birth (which are impossible in reality) and less normally-distributed residuals (94% agreement with a perfect Gaussian distribution as against 97%). Estimates of generalisation error obtained from 5-fold cross validation and 2000 bootstrap samples were within 1% of each other for all models. We therefore report the 8-parameter model

$$\sqrt[{2}]{IB}\;=\;\frac{a+cx+ex^{2}+gx^{3}}{1+bx+dx^{2}+fx^{3}+hx^{4}}$$

where IB denotes inhibin B in pg/mL and x denotes age in years (Fig 1). The parameter values for a, b, …, h are given in Table 2, along with 95% confidence intervals, standard errors and T-values. The residuals for this model are shown in Fig 2; these have 97% agreement with a perfect normal distribution. The coefficient of determination r^2^ = 0.35, indicating that 35% of the variation in inhibin B levels for these ages is due to age alone, with the remaining 65% due to other factors such as genetics, lifestyle and Tanner stage. The repeated imputation of adult inhibin B values had no appreciable effect on the derivation of the model for ages 0–17 years, since the model parameters obtained for each instance were similar (Table 3), being in each case within the 95% confidence intervals for the reported model. The model has a residual mean error of 8.1 pg/ML (square-root adjusted) for the entire dataset. The residual mean training error after 5-fold validation is also 8.1 pg/ML (square-root adjusted), with residual mean test error of 8.3 pg/ML (square-root adjusted).

**Table 2:** Parameter values for the normative model. The 8 parameter values for the model shown in Fig 2, together with their standard error, T statistics and 95% confidence limits.

Parameter	Value	Std Error	T Value	95% CL	95% CL
**a**	**15.4403**	**0.2607**	**59.2154**	**14.9287**	**15.9520**
**b**	**0.8408**	**0.1133**	**7.4214**	**0.6185**	**1.0631**
**c**	**19.6695**	**0.9737**	**20.2015**	**17.7590**	**21.5800**
**d**	**0.4985**	**0.1998**	**2.4954**	**0.1065**	**0.8904**
**e**	**-1.0425**	**1.2055**	**-0.8648**	**-3.4081**	**1.3230**
**f**	**-0.0402**	**0.0140**	**-2.8705**	**-0.0677**	**-0.0127**
**g**	**0.2895**	**0.0682**	**4.2462**	**0.1557**	**0.4233**
**h**	**0.0015**	**0.0003**	**4.9879**	**0.0009**	**0.0020**

**Table 3:** Imputed data analysis. 300 randomly imputed adult data points were added to our combined data before fitting and comparing normative models. This was repeated for a total of 10 imputations, with model parameters collected for comparison and shown in the numbered columns. For any choice of instance, the parameters are within the 95% confidence intervals for each of the remaining instances.

Parameter	1	2	3	4	5	6	7	8	9	10
**a**	**15.4403**	**15.4407**	**15.4392**	**15.4258**	**15.4392**	**15.4388**	**15.4413**	**15.4365**	**15.4384**	**15.4392**
**b**	**0.8408**	**0.8412**	**0.8395**	**0.8235**	**0.8394**	**0.8389**	**0.8418**	**0.8362**	**0.8385**	**0.8394**
**c**	**19.6695**	**19.6666**	**19.6810**	**19.8309**	**19.6817**	**19.6856**	**19.6624**	**19.7099**	**19.6898**	**19.6816**
**d**	**0.4985**	**0.4976**	**0.5017**	**0.5422**	**0.5018**	**0.5029**	**0.4963**	**0.5096**	**0.5041**	**0.5018**
**e**	**-1.0425**	**-1.0482**	**-1.0249**	**-0.8061**	**-1.0241**	**-1.0179**	**-1.0569**	**-0.9816**	**-1.0115**	**-1.0240**
**f**	**-0.0402**	**-0.0404**	**-0.0403**	**-0.0426**	**-0.0403**	**-0.0403**	**-0.0407**	**-0.0406**	**-0.0403**	**-0.0403**
**g**	**0.2895**	**0.2880**	**0.2903**	**0.2906**	**0.2900**	**0.2907**	**0.2853**	**0.2915**	**0.2910**	**0.2903**
**h**	**0.0015**	**0.0015**	**0.0015**	**0.0015**	**0.0015**	**0.0015**	**0.0015**	**0.0015**	**0.0015**	**0.0015**

**Fig 3:**
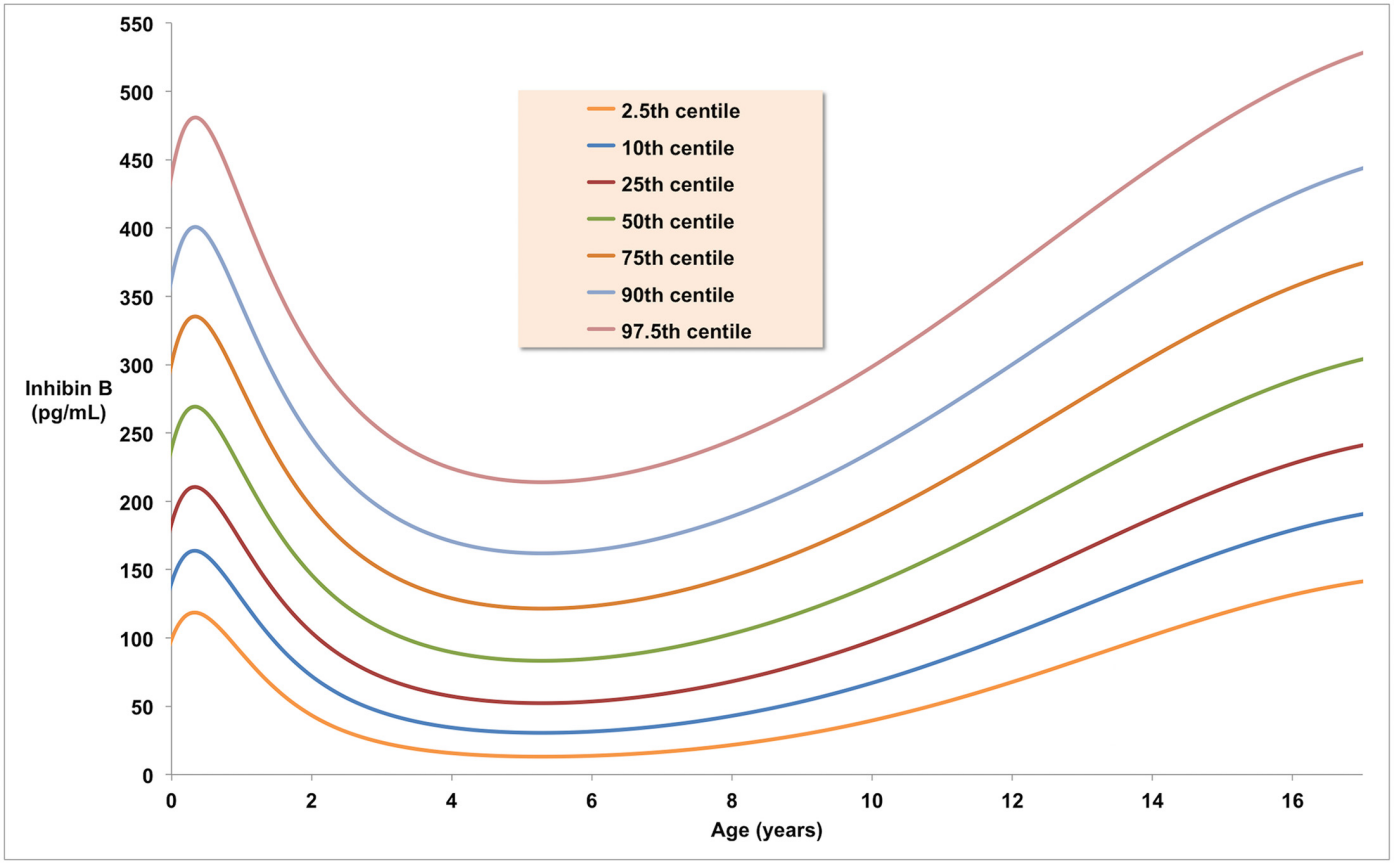
The Normative Model. Inhibin B levels in human males from birth to age 17 years expressed as standard centiles.

Our model shows that inhibin B rises from a mean level of 240 pg/mL (IQR 185–300 pg/mL) at birth in males and reaches a post-natal peak of 270 pg/mL (IQR 210–335 pg/mL) at around 3 months old (Fig 3; Table 4). The level then decreases and remains between 80 and 90 pg/mL (IQR 50–130 pg/mL) for ages 4 to 7 years. Inhibin B begins to rise at around 8 years, peaking at 305 pg/mL (IQR 240–445 pg/mL) at around age 17. Following this peak there is a slow decline to the standard mature adult reference level of 170 pg/mL (IQR 125–215 pg/mL).

**Table 4:** Normative Inhibin B levels in pg/mL. The centiles shown in Fig 3 are here given in tabular form for each year from birth to 17 years.

Age	2.5th centile	10th centile	25th centile	50th centile	75th centile	90th centile	97.5th centile
**0**	**99**	**140**	**183**	**238**	**301**	**362**	**439**
**1**	**89**	**129**	**170**	**223**	**283**	**343**	**418**
**2**	**43**	**72**	**104**	**146**	**196**	**246**	**310**
**3**	**23**	**46**	**71**	**107**	**150**	**195**	**251**
**4**	**16**	**34**	**57**	**90**	**129**	**171**	**224**
**5**	**13**	**31**	**52**	**84**	**122**	**162**	**214**
**6**	**14**	**31**	**53**	**85**	**123**	**164**	**216**
**7**	**17**	**36**	**59**	**92**	**131**	**173**	**227**
**8**	**22**	**43**	**68**	**103**	**145**	**189**	**245**
**9**	**29**	**53**	**81**	**119**	**164**	**210**	**269**
**10**	**40**	**67**	**98**	**139**	**187**	**236**	**299**
**11**	**53**	**84**	**118**	**162**	**214**	**267**	**333**
**12**	**68**	**103**	**140**	**188**	**244**	**300**	**370**
**13**	**85**	**123**	**164**	**216**	**275**	**335**	**408**
**14**	**102**	**144**	**188**	**243**	**306**	**368**	**444**
**15**	**118**	**163**	**209**	**268**	**333**	**398**	**478**
**16**	**132**	**179**	**228**	**289**	**357**	**424**	**506**
**17**	**141**	**191**	**241**	**304**	**374**	**444**	**528**

## Discussion

We have derived a normative model of inhibin B for a healthy human population of males from birth to the age of seventeen. We have shown that, in males, levels of inhibin B rise from birth to a post-natal peak, only to decrease to low, but still measurable, levels during childhood, and increase again from approximately 8 years of age to reach a peak around 17 years old. Our internal validation results suggest that (a) the optimal model is not obtained by a chance selection of suitable data, (b) the reported model is neither over- nor underfitting the data, and (c) the generalisation error when using the model to predict new values is likely to be low.

The finding of a post-natal peak is in keeping with a number of studies. Chada et al, (2003) reported that inhibin B showed a significant increase to a peak at 3–4 months of age and a decrease at 5–11 months [25]. This trend was also a finding by Byrd et al (1998), who showed that males between 13 weeks and 1 year had higher inhibin B levels than post-pubertal males [26]. Our study confirms the finding by Andersson et al (1998) that the post-natal peak exceeds the level of inhibin B found in adult men [7]. The post-natal peak most likely reflects the activation of the hypothalamic pituitary gonadal axis, as the number of Sertoli cells in males during the first year of life can increase fivefold [27]. This is comparable to the increase in AMH that is seen in females during this post-natal period [22]. This postnatal rise may therefore predict later testicular function, in particular spermatogenesis, but longitudinal studies are required to assess that possibility.

These data also show that there is a rise in inhibin B from age 8. Although this analysis does not include pubertal staging, this rise in inhibin B would seem to be earlier than the clinical onset of puberty, likely reflecting prepubertal proliferation of Sertoli cells [28, 29]. The onset of puberty is classically defined by an increase in testicular volume with rising testosterone levels, with mean age of onset at 11.5 years in males [30]: thus the present analysis indicates that there are significant maturational events occurring in the testis before the clinical onset of puberty. The production of significant levels of inhibin B in pre-pubertal males demonstrates that basal inhibin B secretion takes place in the prepubertal testis, despite the very low levels of both gonadotrophins and testosterone [4]. Therefore, one clinical implication of our normative model is its potential use as a reference for a healthy population in assessing the role of inhibin B as a pre-pubertal marker of Sertoli cell function. This has a number of potential clinical implications, including the assessment of functioning testicular tissue in hypogonadotrophic hypogonadism, anorchia and disorders of sexual development [2, 31]. We have reported our experience in assessing inhibin B as a marker of gonadotoxicity in young males treated for malignancy in childhood [11], but the interpretation was limited by the absence of a normative model for inhibin B in childhood.

We have also shown that there is a pubertal peak, as the level at about 17 years old exceeds that of the adult [24]. This is in keeping with a study by Andersson et al (1997), whose results suggest a slight decrease in the level of inhibin B post-pubertally [4]. A study to assess serum inhibin B levels in normal men showed an age-related decline of median inhibin B levels limited to the younger age groups, with stable levels between age 35 and 79 years, and only a modest further decrease thereafter [32].

It is interesting that, in males, inhibin B peaks at a younger age (17 years in our model) than testosterone, which peaks at 19 years [21]. We know from longitudinal studies that spermarche occurs at a median age of 13.4 years (IQR 11.7–15.3 years), with spermatozoa being found in the urine of boys in early puberty [33]. It appears likely that maturation of Sertoli cells to support spermatogenesis can occur in the presence of sub maximal levels of testosterone.

This study has a number of limitations, including its relative small size and different storage procedures for the samples. Crofton et al (2002) reported storing samples for up to eight years at -80°C before analysis [8], but the length of storage is not stated in the other papers. Timing of sample collection varies between studies, however the diurnal variation in serum inhibin B is on average only 3% with higher levels in the morning [34]. This variation is within the limits of the assay accuracy and is therefore unlikely to introduce significant bias. Our treatment of longitudinal data as cross-sectional may have reduced the variability in Inhibin B in our reported model. However, correct variability was enforced for younger ages (i.e. zero Inhibin B at conception) and ages above 17 years (using data imputed from adult reference ranges derived from cross-sectional data), and the use of these values guards against a significant underestimate of the true variability in Inhibin B levels for ages 0–17 years.

In summary we have described a normative model of inhibin B for young males from birth to aged 17 years. We believe this will be an invaluable tool as a reference model for evaluating the role of inhibin B as a marker of Sertoli cell function in the younger male.

## Supporting Information

S1 Dataset Combined Inhibin B data and validation subsets. The combined worksheet has the 709 extracted age-inhibin B pairs, together with imputed values at age 30 and fixed zero values at conception. Raw values in pm/mL are square root adjusted. The Xi columns are the 10-fold imputation variants. The Kj worksheets contain the 80% of the data used for training at each validation fold, together with the 20% remainder used as test data.
(XLS)
